# Evaluation of mineral oil saturated hydrocarbons (MOSH) and mineral oil aromatic hydrocarbons (MOAH) in pure mineral hydrocarbon-based cosmetics and cosmetic raw materials using ^1^H NMR spectroscopy

**DOI:** 10.12688/f1000research.11534.2

**Published:** 2017-08-22

**Authors:** Dirk W. Lachenmeier, Gerd Mildau, Anke Rullmann, Gerhard Marx, Stephan G. Walch, Andrea Hartwig, Thomas Kuballa

**Affiliations:** 1 Chemisches und Veterinäruntersuchungsamt (CVUA) Karlsruhe, Karlsruhe, 76187, Germany; 2 Karlsruher Institut für Technologie (KIT), Institut für Angewandte Biowissenschaften, Lebensmittelchemie und Toxikologie, Karlsruhe, 76131, Germany

**Keywords:** mineral oil, petrolatum, cosmetics, hydrocarbons, polycyclic aromatic hydrocarbons, benzo[a]pyrene, magnetic resonance spectroscopy

## Abstract

Mineral hydrocarbons consist of two fractions, mineral oil saturated hydrocarbons (MOSH) and mineral oil aromatic hydrocarbons (MOAH). MOAH is a potential public health hazard because it may include carcinogenic polycyclic compounds. In the present study, 400 MHz nuclear magnetic resonance (NMR) spectroscopy was introduced, in the context of official controls, to measure MOSH and MOAH in raw materials or pure mineral hydrocarbon final products (cosmetics and medicinal products). Quantitative determination (qNMR) has been established using the ERETIC methodology (electronic reference to access *in vivo* concentrations) based on the PULCON principle (pulse length based concentration determination). Various mineral hydrocarbons (e.g., white oils, paraffins or petroleum jelly) were dissolved in deuterated chloroform. The ERETIC factor was established using a quantification reference sample containing ethylbenzene and tetrachloronitrobenzene. The following spectral regions were integrated: MOSH δ 3.0 – 0.2 ppm and MOAH δ 9.2 - 6.5, excluding solvent signals. Validation showed a sufficient precision of the method with a coefficient of variation <6% and a limit of detection <0.1 g/100 g. The applicability of the method was proven by analysing 27 authentic samples with MOSH and MOAH contents in the range of 90-109 g/100 g and 0.02-1.10 g/100 g, respectively. It is important to distinguish this new NMR-approach from the hyphenated liquid chromatography-gas chromatography methodology previously used to characterize MOSH/MOAH amounts in cosmetic products. For mineral hydrocarbon raw materials or pure mineral hydrocarbon-based cosmetic products, NMR delivers higher specificity without any sample preparation besides dilution. Our sample survey shows that previous methods may have overestimated the MOAH amount in mineral oil products and opens new paths to characterize this fraction. Therefore, the developed method can be applied for routine monitoring of consumer products aiming to minimize public health risks.

## Introduction

Mineral oil hydrocarbons were suggested as important contaminants of the human body, with possible routes of contamination including air inhalation, food intake, and dermal absorption. A correlation was found between the use of cosmetic products, such as creams or lipsticks, and mineral oil saturated hydrocarbons (MOSH) in human fat tissue and in milk samples collected from women ^1^. The mineral oil aromatic hydrocarbons (MOAH) fraction is under scrutiny because it may contain genotoxic carcinogens as contaminants, namely some polycyclic aromatic hydrocarbons (PAH) ^2^. For monitoring of cosmetic products and medicinal products aiming for health risk assessment, a scarcity of analytical methods was noted. This may be explained because the analysis of mineral oil constituents is extremely difficult, and because of their complexity it has been generally unfeasible to resolve the hydrocarbon mixtures into individual components for quantification ^3^. The current method of choice for analysis of hydrocarbons is the application of an online coupled liquid chromatography-gas chromatography with flame ionization detection (LC-GC-FID), which leads to two fractions quantified as sum parameters, MOSH and MOAH ^4^. It is crucial to stress that the acronyms MOSH and MOAH have to be carefully interpreted depending on the actually applied method, e.g. online or offline hyphenated LC-GC-FID or nuclear magnetic resonance (NMR), as described in this paper.

According to the principle of LC-GC-FID, MOSH and MOAH fractions are separated from interfering material by LC and are subsumed according to their retention on the gas chromatographic column. But apart from retention behaviour, the assay includes no information about the chemical properties of the compounds. The resulting chromatograms show regions of overlapped peaks for the MOSH and MOAH fractions (so called humps). Chromatographic integration windows for these humps are empirically identified using n-alkanes as markers. The FID has virtually the same response per unit mass for all saturated hydrocarbons and it is only marginally higher for aromatic hydrocarbons, so that the quantification using cyclohexyl cyclohexane (for MOSH) and 1- and 2-methyl naphthalene (for MOAH) as internal standards is possible ^5, 6^.

Especially for the compounds included in the MOAH region, there is no information about the properties of the detected compounds within the defined retention time windows. Specifically no information about the chain length of substituents or how many polycyclic aromatic rings are included, which may have considerable influence on the toxicity of the compounds ^7^. The critical compounds in the class of polycyclic aromatic hydrocarbons (e.g., the European Food Safety Authority (EFSA) PAH4 group, i.e. benz[ *a*]anthracene, benzo[ *a*]pyrene, chrysene and benzo[ *b*]fluoranthene ^7^) are therefore not specifically determined. It is generally difficult to determine single substances in mixtures of multiple compounds such as mineral oils, which may contain several 1000 of substances. On the other hand, it may also not be appropriate to focus the analysis on only a few single compounds, which may overlook some toxicologically important compounds. For the reason of practicability, it was decided to provide such a total view of MOSH/MOAH sum parameters in mineral oil analysis by hyphenated LC-GC-FID.

Due to the problems in using hyphenated LC-GC-FID techniques, which includes requirements for complex equipment, special training of operators and rather long analysis times, the aim of this study was to evaluate another technique, namely NMR spectroscopy for the purpose of MOSH/MOAH analysis. NMR has so far been only applied to determine the relative proportion of aromatic protons in hydrocarbon resins ^8^ or different fractions in cracked gasoline ^9^, but not for quantification of MOSH/MOAH. In contrast to LC-GC-FID, the analysis of aromatic compounds using NMR may be more precise and selective because the physicochemical properties of the chemical structure of the compound is the underlying criterion for the chemical shifts observed in NMR. Hence the NMR evaluation can be regarded as being much more specific because a chemical property (such as an aromatic ring) is evaluated and not just the retention behaviour of compounds.

## Methods

### Samples and sample preparation

Raw materials intended to be used as ingredients for cosmetics as well as medicinal products were analysed in the capacity of the CVUA Karlsruhe as Official Medicines and Cosmetics Control Laboratory (OMCL/OCCL) for the German Federal State of Baden-Württemberg.

The samples were either provided by the local administrative authorities for routine surveillance or were directly obtained using internet-based mail order. The internet-based sampling also included some technical-quality mineral oil products not intended for use in cosmetics, medicines or foods as comparison samples (listed as ‘Technical products’). An overview of the analysed samples is given in Table 1.

**Table 1.** Results of the quantitative determination of mineral oil saturated hydrocarbons (MOSH) and mineral oil aromatic hydrocarbons (MOAH) by NMR. (n.d. not detectable).

Sample #	Sample description ^a^	Product category	MOAH (g/100 g)	MOSH (g/100 g)	Other compounds (g/100 g)	Benz[ *a*]anthracene (g/100 g)	Benzo[ *a*]pyrene (g/100 g)	Chrysene (g/100 g)	Benzo[ *b*]fluoranthene (g/100 g)
1	Bag balm	Cosmetic product	0.60	100.1	0.09	n.d. <0.23	n.d. <0.13	n.d. <0.11	n.d. <0.13
2	Lip balm	Cosmetic product	0.29	97.5	10.47	n.d. <0.03	n.d. <0.02	n.d. <0.01	n.d. <0.01
3	Bag balm	Cosmetic product	0.99	102.1	0.15	n.d. <0.15	n.d. <0.08	n.d. <0.08	n.d. <0.08
4	Bag balm, yellow	Cosmetic product	0.25	102.1	0.65	n.d. <0.15	n.d. <0.08	n.d. <0.08	n.d. <0.08
5	Vaseline	Cosmetic product	0.56	101.2	0.14	n.d. <0.23	n.d. <0.13	n.d. <0.12	n.d. <0.13
6	Vaseline	Cosmetic product	0.24	101.5	0.01	n.d. <0.03	n.d. <0.02	n.d. <0.02	n.d. <0.02
7	Vaseline	Cosmetic product	0.74	102.7	0.23	n.d. <0.04	n.d. <0.02	n.d. <0.02	n.d. <0.02
8	Vaseline	Cosmetic product	0.64	103.0	0.09	n.d. <0.04	n.d. <0.02	n.d. <0.02	n.d. <0.02
9	Bag balm	Cosmetic product	0.51	99.1	2.63	n.d. <0.03	n.d. <0.02	n.d. <0.02	n.d. <0.02
10	Paraffinum subliquidum	Raw material for cosmetics	0.01	102.8	0.02	n.d. <0.04	n.d. <0.02	n.d. <0.02	n.d. <0.02
11	Paraffinum perliquidum	Raw material for cosmetics	0.02	101.9	0.03	n.d. <0.03	n.d. <0.02	n.d. <0.02	n.d. <0.02
12	Paraffinum perliquidum	Raw material for cosmetics	0.03	105.3	0.08	n.d. <0.04	n.d. <0.02	n.d. <0.02	n.d. <0.02
13	Wax	Raw material for cosmetics	0.13	90.2	0.07	n.d. <0.04	n.d. <0.02	n.d. <0.02	n.d. <0.02
14	Vaseline white Ph. Eur.	Raw material for cosmetics	1.10	95.0	0.11	n.d. <0.04	n.d. <0.02	n.d. <0.02	n.d. <0.02
15	Vaseline white Ph. Eur.	Raw material for cosmetics	0.46	100.4	0.22	n.d. <0.04	n.d. <0.02	n.d. <0.02	n.d. <0.02
16	Liquid paraffin Ph. Eur.	Medicinal product	0.05	103.4	0.17	n.d. <0.04	n.d. <0.02	n.d. <0.02	n.d. <0.02
17	Vaseline white Ph. Eur.	Medicinal product	0.67	102.1	0.21	n.d. <0.06	n.d. <0.03	n.d. <0.03	n.d. <0.03
18	Lamp oil	Technical product	0.11	110.1	0.13	n.d. <0.06	n.d. <0.03	n.d. <0.03	n.d. <0.03
19	Cleaning solvent	Technical product	0.05	108.6	0.04	n.d. <0.06	n.d. <0.03	n.d. <0.03	n.d. <0.03
20	White mineral oil	Technical product	0.02	104.4	0.07	n.d. <0.06	n.d. <0.03	n.d. <0.03	n.d. <0.03
21	Technical Vaseline	Technical product	0.65	103.7	0.25	n.d. <0.06	n.d. <0.03	n.d. <0.03	n.d. <0.03
22	Vaseline yellow Ph. Eur.	Medicinal product	0.81	101.5	0.20	n.d. <0.06	n.d. <0.03	n.d. <0.03	n.d. <0.03
23	Vaseline Triple purification	Technical product	0.40	102.6	0.01	n.d. <0.06	n.d. <0.04	n.d. <0.03	n.d. <0.04
24	Technical petroleum jelly	Technical product	1.04	99.1	0.41	n.d. <0.05	n.d. <0.03	n.d. <0.03	n.d. <0.03
25	Grease for fittings	Technical product	0.13	94.5	0.86	n.d. <0.05	n.d. <0.03	n.d. <0.03	n.d. <0.03
26	Paraffin liquid light	Technical product	0.04	104.5	0.06	n.d. <0.06	n.d. <0.03	n.d. <0.03	n.d. <0.03
27	Paraffin wax powder	Technical product	0.15	108.8	0.21	n.d. <0.06	n.d. <0.03	n.d. <0.03	n.d. <0.03

^a^Products from different brands and manufacturers were analysed if the same product is listed several times (e.g. for bag balm and vaseline).

The samples were prepared for measurement by dissolving ~50 mg of sample in 1.5 mL of CDCl _3_ containing 0.1% tetramethylsilane (TMS) (purity 99%). After membrane filtration using syringe filters with polyester membrane (Chromafil Xtra PET-20/25 0.20 µm, Macherey Nagel, Düren Germany), 600 µl of the solution were poured into an NMR tube for direct measurement.

### NMR method

All ^1^H NMR measurements were performed using a Bruker Ascend 400 spectrometer (Bruker Biospin, Rheinstetten, Germany) equipped with a 5-mm SEI probe PA BBI 400S1 with Z-gradient coils and a Bruker automatic sample changer (Sample Xpress, Bruker Biospin). All spectra were acquired at 300.0K.

NMR spectra were acquired using the Bruker standard zg30 pulse sequence with 32 scans and 2 prior dummy scans (DS). The acquisition parameters were based on a previously described method ^10^.

All spectra were recorded in the *baseopt* mode (generates a smooth baseline at zero without offset). The acquisition parameters were kept constant for reference and sample spectra for PULCON measurements according to Monakhova *et al*. ^11^. For each sample during spectra acquisition, the 90° pulse width (P1 in Bruker terminology) was set at 8 ms, the sweep width (SW) was 20.5504, and the size of the real spectrum (SI) was 131072.

The data were acquired automatically under the control of ICON-NMR 5.0.6 (Bruker Biospin), requiring 25 min per sample including temperating. All NMR spectra were phased, baseline-corrected and manually integrated using Topspin 3.2 (Bruker Biospin).

### Quantification using the PULCON principle

The application of PULCON (PULse length based CONcentration determination) quantification was previously described in detail ^11^. In short, the ERETIC (Electronic REference To access *In vivo* Concentrations) factor was calculated for each measurement series based on a so-called quantref sample measured as the first sample in the series. The quantref sample was prepared by dissolving 50 mg tetrachloronitrobenzene and 50 mg ethylbenzene in 10 mL of CDCl _3_ containing 0.1% TMS. The ERETIC factor was determined using the following equation:

$$ERETIC=\frac{I\cdot MW}{C\cdot N}(\text{eq}.1)$$

where I is the absolute integral, MW is the molecular weight (260.89 for tetrachloronitrobenzene or 106.17 for ethylbenzene), N is the numbers of protons generating the selected signal, and C is the concentration. The following signals were integrated: tetrachloronitrobenzene: δ 7.83-7.64 ppm (N=1, s); ethylbenzene δ 7.23-7.11 ppm (N=3, m), δ 2.75-2.54 ppm (N=2, q), δ 1.35-1.13 ppm (N=3, t). The average ERETIC factor of all four signals was used for further calculations.

The analyte concentrations in the samples were then calculated using the following equation:

$$C=\frac{I\cdot MW}{ERETIC\cdot N}\;(\text{eq}.\;2)$$

where I is the absolute integral, MW is the molecular weight (128.17 for naphthalene, 138.25 for decalin, 92.09 for glycerol, 228.29 for benz[ *a*]anthracene, 252.32 for benzo[ *a*]pyrene, 228.29 for chrysene, and 252.32 for benzo[ *b*]fluoranthene), ERETIC is the eretic factor (see eq. 1) and N is the numbers of protons generating the selected signal.

**Figure 1.**
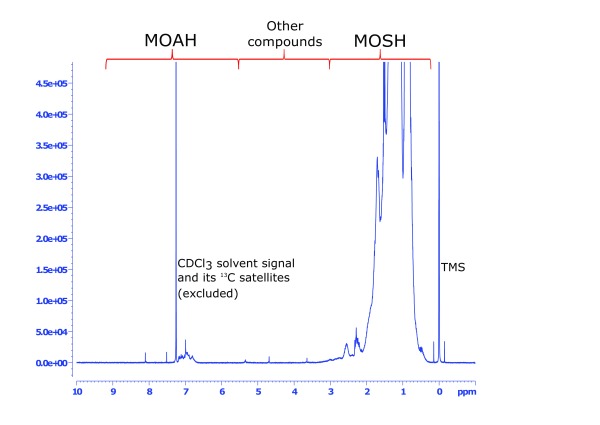
Overview of the complete ^1^H NMR spectrum of sample #1 (bag balm), showing the spectral regions used for MOSH and MOAH integration. MOAH, mineral oil aromatic hydrocarbons; MOSH, mineral oil saturated hydrocarbons; TMS, tetramethylsilane.

The integral ranges were chosen as follows: MOSH region calculated as decalin equivalents: δ 3.0-0.2 ppm (N=18) except the regions of residual H _2_O around δ 1.53 ppm; MOAH region calculated as naphthalene equivalents (N=8): δ 9.20–7.55, δ 7.50–7.30, δ 7.22–7.00, and δ 6.97–6.50 (the three regions cut out are due to the residual undeuterated solvent signals of CHCl _3_ and its ^13^C satellites); and region of other compounds calculated as glycerol (N=5): δ 6.50-3.00. The integral regions are also shown in Figure 1. For targeted quantification of the EFSA PAH4 group, the following integration regions were used: benzo[ *a*]pyrene δ 9.06–8.98 (N=2), chrysene δ 8.76–8.70 (N=2), benz[ *a*]anthracene δ 9.20–9.12 (N=1) and benzo[ *b*]fluoranthene δ 7.45–7.38 (N=2).

To check the quality of the value of the ERETIC factor in terms of precise initial balance, sample preparation and NMR experiment, the concentration in a control solution (cyclohexane, 2500 mg l ^-1^, CDCl _3_, singlet at δ 1.53–1.36 ppm, N = 12) was measured at the end of each measurement series. The recovery had to be 100 ± 5% to perform further calculations for the samples. The limit of detection (LOD) was manually estimated based on a small, but still integratable, signal in the region of PAH4 ^12^.

## Results

**Figure 2.**
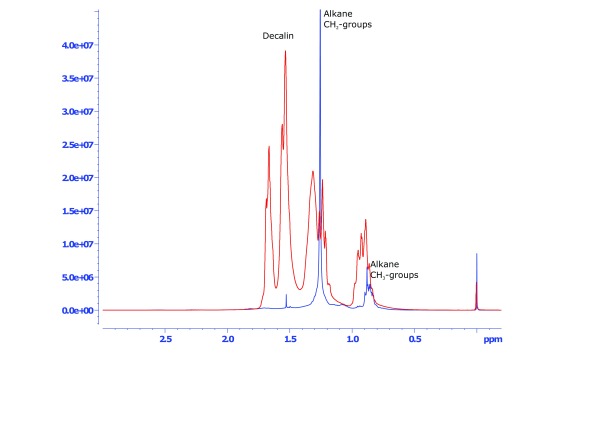
NMR spectra of the region 3.0-0.2 ppm containing the mineral oil saturated hydrocarbons (MOSH) of sample #1 (blue line) in comparison to the standard substance decalin (red line).

**Figure 3.**
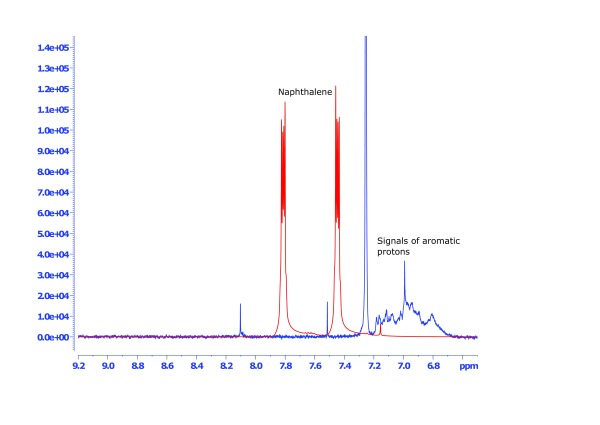
NMR spectra of the region 9.2-6.5 ppm containing the mineral oil aromatic hydrocarbons (MOAH) of sample #1 (blue line) in comparison to the standard substance naphthalene (red line).

An overview of a typical NMR spectrum of a mineral oil-based cosmetic product (final cosmetic product based on pure petroleum jelly) is shown in Figure 1. Both MOSH and MOAH regions show considerable signals, which are however very overlapped due to the multi-mixture character of mineral oils. The MOSH fraction is much more abundant than the MOAH fraction, which is obvious by the much larger signal range between δ 3 and 0.2 ppm. In the MOSH region (magnification in Figure 2), the specific signals for alkane-type CH _2_ and CH _3_ groups are separable. In the MOAH region (magnification in Figure 3), such a structural assignment appears not possible as the aromatic protons in the region of δ 7.2 to 6.8 show a much more overlapped behaviour. In the middle of the spectrum, around 4 ppm ( Figure 1), a region remains that includes chemical structures not characteristic to either MOSH or MOAH definitions. The region was therefore separately quantified ( Table 1, column “other compounds”), and may provide evidence about the magnitude of admixture of non-mineral oil ingredients in the cosmetic formulations.

In addition to the quality control measure of a control sample in each series, which showed in all cases a coefficient of variation below 5%, a validation by spiking of standard substances into a paraffin oil sample has been conducted ( Table 2). The validation results show acceptable recoveries typically between 97 and 102%, but only at the edges of the working range, a higher imprecision was observed (84–111%). The average coefficient of variation (CV) of the validation measurements was 6.1%. Additionally, a reference solution of chrysene (0.55 g/100 g) was measured 6 times with a CV of 2.9% and an average recovery of 99.7%. Finally, the CV of the control sample determined over 10 measurement days was 2.8% and the average recovery was 99.5%. The LOD of the method for PAH4 is in the range of 0.01-0.4 g/100 g (depending on sample weight and spectral background).

**Table 2.** Method validation results obtained by spiking reference standards on authentic paraffin oil samples.

	Validation Sample 1	Validation Sample 2	Validation Sample 3	Validation Sample 4	Validation Sample 5	Validation Sample 6
Spiked concentration naphthalene [g/100 g]	1.01	2.04	3.04	4.05	5.07	5.07
Spiked concentration decalin [g/100 g]	11.2	22.3	33.5	44.7	55.8	55.8
Measured concentration naphthalene [g/100 g]	0.94	1.99	2.94	3.94	5.00	5.17
Measured concentration decalin [g/100 g]	9.3	22.3	32.9	43.6	56.6	62.1
Recovery naphthalene [%]	92.7	98.2	96.7	97.2	98.7	102.0
Recovery decalin [%]	83.6	99.9	98.2	97.5	101.3	111.3

Table 1 shows the quantitative results of 27 samples. Most of the samples were vaseline or petroleum jelly type products, which are offered either as cosmetic raw materials, cosmetic products or medicinal products according to EU-Pharmacopeia quality or in a technical grade quality. MOSH and MOAH were quantifiable in all samples. The highest MOAH amounts of 1.10 g/100g and 1.04 g/100 g were found in a vaseline raw material for cosmetics and a technical grade quality vaseline, respectively. The lowest amount of 0.02 g/100 g was detected in a product placed on the market as a final cosmetic product. Liquid mineral oil products were found to have lower MOAH amounts (typically less than 0.1 g/100 g) than solid products.

The MOSH amount in all samples iterated around 100% as expected in almost pure mineral oil products. The amount of other compounds besides MOSH and MOAH was also generally very low (<1%), with some exceptions of compounded products, such as lip balm or bag balm, which contain other ingredients besides mineral oils. In none of the products were any of the PAH4 group compounds detectable.

NMR raw data are provided as JCAMP-DX files in a zipped file Type of archive file: JCAMP DIFF/DUP with included data types FID+RSPEC+ISPEC; JCAMP version 6.0. The software Topspin 3.2 (Bruker Biospin) was used for data export. The data include the raw and processed spectra of five measurement series including the 27 samples as well as quantref and control samples and spectra of standard substances measured for comparison. Click here for additional data file. Copyright: © 2017 Lachenmeier DW et al. 2017 Data associated with the article are available under the terms of the Creative Commons Zero "No rights reserved" data waiver (CC0 1.0 Public domain dedication).

## Discussion

The advantages of NMR are the simple sample preparation, which only consists of diluting 50 mg of sample in solvent followed by membrane filtration, and the short measurement time. The measurement time per sample is about 25 min including temperating. NMR is focussed on the actually contained aromatic amounts and hence the results appear to be suitable for toxicological assessment.

The method validation results were similar to other NMR methods based on PULCON quantification ^11, 13– 15^ and were judged as acceptable for the application of the method for official cosmetics control purposes ^16^. The LOD of the method was also in agreement with the LOD of <0.1% reported for protons of olefins (δ 4.5-6.7 ppm) ^8^.

An advantage of NMR is that a full automation (including all steps of spectra processing, integration and calculation) is possible due to the very simple approach of PULCON quantification ^17^. This in combination with the very rapid measurement of NMR allows a very high sample throughput in routine analysis. In the current method development and validation work, the spectra were evaluated only semi-automatically (meaning integration by manual operation of each spectrum). We therefore expect that improved method validation data might be achievable when fully automated spectral processing and integration will be implemented in the next step of routine application of the method.

In comparison to LC-GC-FID, for which the sum of both fractions (MOSH+MOAH) seldom leads to 100%, it is plausible that this is possible with NMR. During LC-GC-FID only a certain part of the sample is considered by the cutting of certain fractions from the first column to the second column. Additionally, certain integration regions are selected during LC-GC-FID so that not all eluting compounds are included. This corroborates findings by Lommatzsch *et al*. ^8^, who reported that differences between NMR and LC-GC-FID occur because hydrocarbons of higher molecular weight are not determined by the latter method (leading to lower MOSH amounts in LC-GC-FID than in NMR) and because the saturated part of the molecule is included in the quantification as well (leading to higher MOAH amounts in LC-GC-FID than in NMR). NMR can also simultaneously quantify the range of miscellaneous compounds not belonging to the MOSH/MOAH fractions.

**Figure 4.**
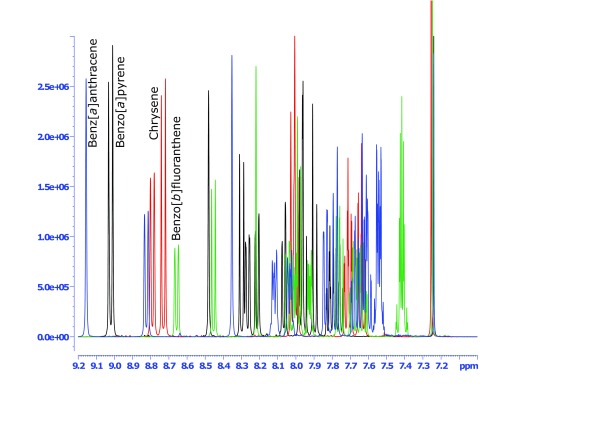
NMR spectra of standard solutions of the PAH4 group compounds benzo[ *a*]pyrene (black line), chrysene (red line), benz[ *a*]anthracene (blue line) and benzo[ *b*]fluoranthene (green line).

A further advantage of NMR quantification is the use of a specific compound for quantification. Naphthalene-equivalents were chosen as a marker to determine the MOAH fraction, as this is the approach of the European Pharmacopeia method for quantification of total polycyclic aromatic hydrocarbons using UV spectrophotometry ^18^. As a saturated counterpart of naphthalene, we have chosen decalin-equivalents for the MOSH fraction. Decalin is also expected to correspond to the average of aliphatic compounds in mineral oils. However, like in all other techniques applying an index based on single compounds, and due to the variations in composition of mineral hydrocarbons (white oils, petrolatum, microcrystalline waxes, ozokerites, ceresines and paraffines), it can be explained that some samples had MOSH results of over 100%. While NMR clearly gives better selectivity for aromatic structures, similar limitations as in LC-GC-FID have to be accepted, meaning that a complete spectral region is quantified and defined as MOSH or MOAH. This information initially also does not include information about the amount of polycyclic rings. As we have clearly shown that the most relevant signals of PAH compounds are part of the aromatic region ( Figure 4), the analysis at least provides information about the maximal possible amount of PAH, which would be much lower in reality. Otherwise, the sensitivity of NMR is unsuitable for a targeted quantification of PAH, which are typically expected to occur at amounts of considerably less than 1 mg/kg.

As discussed before, the results of our approach and our sample collection show deviations to previous LC-GC-FID results, especially in the toxicologically relevant aromatic region, which were much higher than the NMR results. There is a general lack of literature about MOSH/MOAH contents in cosmetic raw materials. However, compared to the few studies available, our MOAH results are much lower than some data reported by the German Federal Institute for Risk Assessment (BfR) ^19^ with about 1.7–5.0% MOAH in commercial cosmetic products based on petroleum jelly. The results of this study are more consistent with data from Niederer ^20^ that reported a MOAH range of 0.05–4.5% (average 1.2%) in 38 paraffin oils contained in lip-gloss products, but our average is still lower at <1%. Moret *et al.* assumed that 70–80% of mineral oil is MOSH (depending on the source and the raffination process) ^21^, however, the composition of the remaining 20–30% were unspecified but not assumed as being MOAH (see referee report of Conte & Moret below). In contrast, our results from market samples show that mineral oils consist of almost 100% MOSH. The discrepancies to previous studies may be interpreted not only by methodological differences, but also by the different sample collectives. We are fully aware that mineral oil contaminants in food from food packaging or other sources have to be strictly distinguished from highly refined mineral oils used as raw materials for cosmetic or medicinal products. Nevertheless, the described discrepancy in terms of the MOSH and MOAH amounts points out the controversy mentioned in the introduction about the definition of MOSH/MOAH and suggests that the results must be carefully interpreted depending on the sample source (contaminant, matrix) and method.

A limitation of using NMR for compounded cosmetics is that the MOAH region is not completely specific for mineral oil aromatic compounds, but may include some other aromatic ingredients as well as certain non-aromatic compounds, such as formic acid and its ester. Therefore, the method is not directly suitable without modifications for compounded cosmetic products (e.g. lipsticks, skin care products), which may contain aromatic ingredients, such as UV filters, preservatives, perfuming or colouring agents. These substances may also contain signals in the aromatic region and therefore lead to an overestimation of MOAH. For these reasons, the current work was mostly focused on pure mineral oil products and raw materials. Future research will include sample preparation steps to separate the MOAH fraction from these other ingredients. For the current study, all these interfering compounds were not expected in most of the researched products, therefore we believe that our NMR method gives reliable results. If such interfering compounds are to be expected in cosmetic products, some form of sample preparation (e.g. clean-up using solid-phase extraction) has to be conducted, and the current results need to be interpreted as tentative and potential overestimations.

We observed another limitation during preliminary trials with longer chain compounds (wax-like samples), which were not completely soluble in CDCl _3 _so that under-quantification can be expected for MOSH. This effect has probably no influence on the more toxicologically relevant MOAH compounds, which should be well soluble in CDCl _3_^22^.

A further limitation is the sensitivity of NMR. This seems to be sufficient for the detection of MOSH and MOAH, but the LOD may be too high for a trace analysis of specific PAH. Benzo[ *a*]pyrene is limited in mineral hydrocarbon raw materials to 0.005% (w/w) (Annex II number 620 ff. of regulation (EC) No 1223/2009; http://eur-lex.europa.eu/legal-content/EN/TXT/?uri=CELEX:02009R1223-20170303). However, it is also difficult with any other technique to conduct targeted quantification of single compounds in the complex mixture of mineral oil.

## Conclusions

The presented method is fit-for-purpose to obtain monitoring data of MOSH/MOAH in raw materials or pure mineral hydrocarbon-based cosmetic products, aimed at market surveillance and public health evaluation.

Due to the higher specificity and selectivity of NMR, it must be assumed that the LC-GC approach may have been overestimated the MOAH fraction by possible co-elution of compounds that have similar retention behaviour, but do not contain aromatic ring systems. We therefore conclude that – at least for the product groups of cosmetics and medicinal products, which are based on more or less pure hydrocarbons (petroleum jelly) – LC-GC results should be complemented by the NMR-approach (or an alternative confirmatory technique such as GC coupled with mass spectrometry ^23^ or LC coupled with two-dimensional GC ^24^) to perform a suitable risk assessment.

Nevertheless, risk assessment of MOSH/MOAH in cosmetic products will be a huge challenge in the light of the sum parameter character of these compound groups. The NMR-approach appears to provide an important piece of the puzzle. It is extremely suitable for rapid screening of mineral oil raw materials in terms of MOSH/MOAH. The BfR concluded, based on LC-GC-FID analyses, that MOAH amounts in mineral oil in the percentage range (>1%) are technically avoidable, with the potential to be further reduced to trace amounts ^19^. To substantiate this conclusion on the basis of a statistically representative data set, it is important to firstly develop reliable and reproducible methods, according to article 12 of the EU cosmetics regulation EC/1223/09, and secondly conduct market monitoring.

Such a market surveillance study of the available mineral hydrocarbons for cosmetics is therefore important to obtain representative data. Mineral hydrocarbon raw materials for cosmetic and medicinal products have to be analysed to get a complete view about statistically founded orientation values for MOAH. Additionally, a specific and sensitive chromatographic method for the determination of PAH4 appears to be necessary to characterize toxic polycyclic aromatic compounds. Further toxicity studies are necessary, as well as epidemiological studies that need to confirm that MOSH concentrations may accumulate in human fat tissue, with cosmetics being a potentially relevant source of the contamination ^1, 2, 25, 26^.

## Data availability

The data referenced by this article are under copyright with the following copyright statement: Copyright: © 2017 Lachenmeier DW et al.

Data associated with the article are available under the terms of the Creative Commons Zero "No rights reserved" data waiver (CC0 1.0 Public domain dedication). 

**Dataset 1: NMR raw data are provided as JCAMP-DX files in a zipped file.** Type of archive file: JCAMP DIFF/DUP with included data types FID+RSPEC+ISPEC; JCAMP version 6.0. The software Topspin 3.2 (Bruker Biospin) was used for data export. The data include the raw and processed spectra of five measurement series, including the 27 samples, as well as quantref and control samples and spectra of standard substances measured for comparison. doi, 10.5256/f1000research.11534.d161209 ^27^
