# Supplementary material for: Evaluation of mineral oil saturated hydrocarbons (MOSH) and mineral oil aromatic hydrocarbons (MOAH) in pure mineral hydrocarbon-based cosmetics and cosmetic raw materials using 1H NMR spectroscopy
Source: F1000Res. 2017 Aug 22;6:682. Originally published 2017 May 16. [Version 2] doi: 10.12688/f1000research.11534.2 (PMC5497826; doi:10.12688/f1000research.11534.2)
Supplement: Peer review history

# Peer review: Referee response for version 2

**DOI:** 10.5256/f1000research.13471.r25257

**Published:** 2017-08-22

**Authors:** Lanfranco S. Conte, Sabrina Moret

**Affiliations:** 1 Department of Food Science, University of Udine, Udine, Italy; 2 Department of Agro-Food, Environmental and Animal Sciences, University of Udine, Udine, Italy

The paper had been modified, where needed and some concerning aspects had been adequately modified and stressed.

 In this form, the paper is suitable for indexing.

We have read this submission. We believe that we have an appropriate level of expertise to confirm that it is of an acceptable scientific standard.

# Peer review: Referee response for version 1

**DOI:** 10.5256/f1000research.12458.r23855

**Published:** 2017-06-28

**Authors:** Richard H. Stadler

**Affiliations:** 1 Institute of Food Safety and Analytical Sciences, Nestlé Research Center, Lausanne, Switzerland

The authors present a method using NMR to determine MOSH and MOAH in cosmetics and cosmetic raw materials. Overall this work is important, as it provides a complementary approach to screening methods such as GC-FID. I have only a few comments that the authors are invited to comment on:

- Clear deviations of NMR versus LC-GC-FID results are described in the paper, for example MOAH amounts are lower by the NMR method. However, no direct comparison on the same sample is reported, which would strengthen their argument.
- The need for a confirmatory approach in foods and food raw materials, albeit using GC-MS, has recently been reported by Spack *et al* in Food Additives & Contaminants, 2017 ( http://dx.doi.org/10.1080/19440049.2017.1306655). This reference should be included on page 10, after the paragraph “…. LC-GC results should be complemented by the NMR-approach **(or alternative confirmatory technique such as GC-MS)** to perform a suitable risk assessment,……..”.
- I am not certain if NMR is the appropriate methodology to determine PAH4, as it will not achieve the required sensitivity / specificity, so less focus should be placed on these compounds. I would suggest that the authors rather identify specific aromatic hydrocarbon markers to possibly identify the origin of the “contamination”, which would be a next step in the research approach.

I have read this submission. I believe that I have an appropriate level of expertise to confirm that it is of an acceptable scientific standard.

# Response

**Published:** 2017-08-16

**Authors:** Dirk Lachenmeier

**Affiliations:** Chemisches und Veterinäruntersuchungsamt (CVUA) Karlsruhe, Germany

We thank the referee for his insight and providing suggestions to improve our paper.

1. Regarding direct comparisons between LC-GC-FID and NMR, the referee is correct that direct comparisons of same samples would be preferable. Actually, we have recently purchased the necessary LC-GC-FID equipment, but are still establishing and validating the procedure, which was more challenging than expected. At the current stage, we can therefore not provide the data comparison for all our samples. However three samples, which we had obtained from another laboratory with LC-GC-FID capabilities, confirmed our assumptions. The LC-GC-FID MOAH was 0.8-6.9% while the NMR MOAH was 0.3-1.0%. The LC-GC-FID MOSH was 53-61% while the NMR MOSH was 101-102%. These limited results strengthen our assumption that LC-GC-FID may overestimate MOAH and underestimate MOSH. However, much more samples are needed for a robust conclusion.
2. Thank you for the suggestions. The text suggestion and reference were added as requested.
3. The referee is right that NMR is not appropriate to determine PAH4 due to the required sensitivity (e.g. the DIN EN 16143 procedure using double LC cleaning and GC/MS analysis has a working range of 0.1-15 mg/kg for petroleum products, which cannot be reached by NMR). Nevertheless, NMR would offer the selectivity to distinguish the PAH4 if they should occur at levels above about 0.01%. A sentence was added to the discussion that NMR is not offering sensitivity for targeted quantification of PAH.

# Peer review: Referee response for version 1

**DOI:** 10.5256/f1000research.12458.r23439

**Published:** 2017-06-13

**Authors:** Lanfranco S. Conte, Sabrina Moret

**Affiliations:** 1 Department of Food Science, University of Udine, Udine, Italy; 2 Department of Agro-Food, Environmental and Animal Sciences, University of Udine, Udine, Italy

The submitted manuscript deals with evaluation of MOSH&MAOH that is nowadays an hot topic mainly for foods; a new approach for MOSH and MOAH determination in pure mineral oil based cosmetics and cosmetic raw materials using ^1^H NMR spectroscopy is described.

 The NMR methods seem to be standard ones and well established, and already implemented in many instruments even if for different purposes.

 The technique used seems promising even though, as stated by the author it suffers of approximation: MOAH data are expressed as naphthalene-equivalents, while as known MOAH include a complex mixture of alkylated aromatics, mainly (mono- di- and tri-aromaticis). Maybe a better characterization of these different classes of aromatics could help in reaching better results.

 As known, PAHs are not typically of petrogenic origin, but mainly originate from combustion processes, so strictly speaking they should not be considered as MOAH (even though they can be present in trace amount in MOH mixture). This should be clarified in the introduction.

 The author compare results obtained with NMR with those obtained with on-line LC-GC by other authors, but a direct comparison on the same samples is lacking. A comparison with GCxGC data could be also very interesting. Furthermore,  it seems worthwhile to check if the sensitiveness  of the NMR method meets the requirement usually request for this kind of contaminants.

 Reference 21 is not cited in a pertinent way. The paper cited deals with optimization of a rapid  SPE-GC-FID method for MOSH determination in vegetable oil (not suitable for MOAH determination) and it  doesn’t report that  70-80% of mineral oil in olive oil is MOSH, letting to intend that the remaining 20-30% is MOAH (fortunately this is not true). Concerning MOSH content the author added…”Our results from the market samples show that this is almost 100%”. It is not clear if they refer to cosmetics or olive oil. In the latter case they should report the reference or show the data.

 The paper needs some revision before being reconsidered for indexing.

We have read this submission. We believe that we have an appropriate level of expertise to confirm that it is of an acceptable scientific standard, however we have significant reservations, as outlined above.

# Response

**Published:** 2017-06-19

**Authors:** Dirk Lachenmeier

**Affiliations:** Chemisches und Veterinäruntersuchungsamt (CVUA) Karlsruhe, Germany

Thank you for reviewing our paper. To facilitate our revision, could you please clarify the following point:

The paper in Ref. 21 reports: "The method only determines the MOSH, which is usually

around 70–80% of the mineral oil (depending on the source and the raffination)." (p. 1902, right column, 2nd paragraph of conclusion). We therefore believe that we have not misrepresented the reference and also had no intention to imply indirectly that the remaining 20-30% are MOAH (we will clarify this, of course, during our revision of the paper).

However, we also believe that the 70-80% MOSH does not refer to olive oils but to pure mineral oils in general, and this was the reason that we included this statement, which is one of the few quantitative data in the literature available for comparison. Unfortunately, Ref. 21 does not report any reference for the claim of 70-80% MOSH.

Could you please clarify your intended meaning in Ref. 21 if this range refers to mineral oils in olive oils or to mineral oils in general, before we try to improve the text of our paper.

# Response

**Published:** 2017-08-16

**Authors:** Dirk Lachenmeier

**Affiliations:** Chemisches und Veterinäruntersuchungsamt (CVUA) Karlsruhe, Germany

We thank the reviewers for their constructive comments regarding our study.

1. As requested, it was clarified in the introduction that PAHs are not typical constituents of MOAH but may occur as contaminants in this fraction.
2. Regarding comparison with chromatographic methods, see answer above in response #1 to Richard Stadler. Additionally, we want to stress that the sensitivity of NMR is absolutely sufficient for quantification of the MOSH and MOAH fractions (all samples were positive). The sensitivity may only be insufficient for specific quantification of single PAHs, which now has been clarified in the discussion.
3. While we believe that we had correctly cited Ref. 21 (see also comment above) and no clarification from the reviewers was received in the meantime, we have changed the sentence in the discussion to hopefully make this point much clearer, including a specific statement that the remaining 20-30% are not MOAH.

# Peer review: Referee response for version 1

**DOI:** 10.5256/f1000research.12458.r22775

**Published:** 2017-06-01

**Authors:** Markus Niederer

**Affiliations:** 1 State Laboratory Basel-City, Basel, Switzerland

In this manuscript, the authors present a new application of NMR to measure MOSH and MOAH in raw materials or in final products (e.g. cosmetics). They present a carefully performed validation of the method and compare the NMR-results with those of established LC-GC methods. Especially, the findings of a possible overestimation of the MOAH fraction or underestimation of the MOSH-fraction by LC-GC seem to be very important in terms of risk assessment. Further, advantages and limitations of NMR are presented very detailed and in an objective manner.

 Indexing of the article is recommended because of novelty and technical quality. This paper will be of interest to many scientists in this field of research, especially for toxicological evaluations and for market survey purposes of cosmetic and medicinal products.

 In my opinion, this manuscript is clearly presented and well organised. It gives adequate references to related work and the abstract provides a quantitative summary.

 Remarks:

1. The discussion section could be supplemented with the possibilities of LC-GCxGC-MS in order to characterise components of MOAH.
2. Discussion, Page 9, last section: *“Due to the higher specificity and selectivity of NMR, it must be assumed… We therefore conclude that …”*
  Please move these statements from the discussion to the conclusion section.

I have read this submission. I believe that I have an appropriate level of expertise to confirm that it is of an acceptable scientific standard.

# Response

**Published:** 2017-08-16

**Authors:** Dirk Lachenmeier

**Affiliations:** Chemisches und Veterinäruntersuchungsamt (CVUA) Karlsruhe, Germany

We would like to thank the reviewer for his time and constructive feedback.

1. As requested the discussion was supplemented with mentioning the possibilities of LC-GCxGC-MS.
2. The section was moved from the discussion to the conclusion as requested.
